# Draft Genome Sequences of *Tremellomycetes* Strains Isolated from the International Space Station

**DOI:** 10.1128/MRA.00504-20

**Published:** 2020-06-25

**Authors:** Swati Bijlani, Nitin K. Singh, Christopher E. Mason, Clay C. C. Wang, Kasthuri Venkateswaran

**Affiliations:** a University of Southern California, Los Angeles, California, USA; b Jet Propulsion Laboratory, California Institute of Technology, Pasadena, California, USA; c The WorldQuant Initiative for Quantitative Prediction, Weill Cornell Medicine, New York, New York, USA; University of Maryland School of Medicine

## Abstract

The draft genome sequences of six eukaryotic microbial strains belonging to the class *Tremellomycetes* isolated from the International Space Station were assembled. Further characterization of these sequences will aid in the understanding of the influence of microgravity conditions on these organisms’ potential pathogenicity.

## ANNOUNCEMENT

In an ongoing microbial observatory experiment on the International Space Station (ISS), species belonging to the class *Tremellomycetes* were identified (1). This class comprises yeasts, dimorphic fungi, and organisms that form hyphae or complex fruiting bodies (2). In this class, the genera *Naganishia* and *Papiliotrema* were amended to accommodate the Cryptococcus albidus clade and a few other *Cryptococcus* species, respectively (3). Among the *Cryptococcus* species, Cryptococcus neoformans and Cryptococcus gattii are the most common human pathogens; however, recently, there has been an increase in infections caused by non-*neoformans Cryptococcus* species (4–8). This report presents the draft genome assemblies of four such non-*neoformans Cryptococcus* species, enabling the identification of genetic determinants responsible for their potential pathogenicity under the influence of microgravity compared to their ground controls.

In this study, the draft genome sequences of six strains belonging to the class *Tremellomycetes* isolated from the ISS were determined (1). Descriptions of the sample collection, processing, and presumptive identification of these isolates were published elsewhere (1). Briefly, samples collected from the ISS were processed, and 100 μl of each dilution was plated onto potato dextrose agar (PDA) with 100 μg/ml chloramphenicol. The plates were incubated at 25°C for 7 days. The single colony obtained was restreaked onto PDA plates and incubated at 25°C for 3 days, and a biomass of approximately 1 μg wet weight was collected and pooled for DNA extraction. Total nucleic acid extraction was carried out using a ZymoBIOMICS 96 MagBead DNA kit (Lysis tubes) (Zymo Research, USA) after bead beating using a Bertin Precellys homogenizer. This was followed by library preparation using the Illumina Nextera Flex protocol as per Illumina document number 1000000025416 v07. The initial amount of DNA for library preparation was quantified, and depending on the input DNA concentration, 5 to 12 cycles of PCR were carried out to normalize the output. The amplified genomic DNA fragments were indexed and pooled in a 384-plex configuration. Whole-genome shotgun sequencing was performed on a NovaSeq 6000 S4 flow cell paired-end (PE) 2 × 150-bp platform with a paired-end module. The data were filtered with the NGS QC Toolkit v2.3 (9) for high-quality (HQ) vector- and adaptor-free reads for genome assembly (cutoff read length for HQ, 80%; cutoff quality score, 20). The numbers of filtered reads obtained are listed in Table 1, and they were used for assembly with the SPAdes v3.14.1 (10) genome assembler (k-mer size, 32 to 72 bases). Default parameters were used for all software. The details of the final assembly are summarized in Table 1.

**TABLE 1:** Summary of the draft whole-genome sequences of six strains belonging to the class *Tremellomycetes* isolated from the ISS

Species and strain	NCBI accession no.	Isolation location	No. of scaffolds	Genome size (bp)	*N*_50_ (bp)	Median coverage (×)	G+C content (%)	No. of filtered reads used for assembly (million)
*Naganishia adeliensis* IF1SW-F1	JAAZPZ000000000.1	Port panel next to cupola	149	19,403,212	506,784	134	53.37	14.71
Naganishia albida IF6SW-B1	JAAZPV000000000.1	PMM port 1^*a*^	128	19,422,953	506,783	303	53.37	34.76
*Naganishia* sp. IF7SW-B1	JAAZQA000000000.1	Lab overhead 3	123	19,429,219	511,828	286	53.37	32.79
*Naganishia albida* IIF5SW-F1	JAAZPY000000000.1	Node 1 overhead 4	136	19,428,531	506,790	258	53.37	30.81
Papiliotrema laurentii IF7SW-B5	JAAZPW000000000.1	Lab overhead 3	155	19,200,339	389,502	234	56.19	27.19
*Papiliotrema laurentii* IF7SW-F4	JAAZPX000000000.1	Lab overhead 3	128	19,020,389	484,400	270	56.22	30.21

a PMM port 1, permanent multipurpose module.

The species were identified based on the internal transcribed spacer (ITS) sequences extracted from the assembled genomes. The ITS sequence of *Naganishia* sp. strain IF7SW-B1 retrieved from the assembled genome did not show ≥98% identity to any *Naganishia* species and, therefore, requires taxonomic characterization. Isolation of *Naganishia* and *Papiliotrema* species from the ISS is significant, and their persistence during space flight needs to be further studied.

### Data availability.

The whole-genome sequences and raw data have been deposited in GenBank under the BioProject accession number PRJNA623412. This project has also been deposited in the NASA GeneLab system (GLDS-290; https://genelab-data.ndc.nasa.gov/genelab/accession/GLDS-290). The version described in this paper is the first version.
